# Attitudes and Stereotypes in Lung Cancer versus Breast Cancer

**DOI:** 10.1371/journal.pone.0145715

**Published:** 2015-12-23

**Authors:** N. Sriram, Jennifer Mills, Edward Lang, Holli K. Dickson, Heidi A. Hamann, Brian A. Nosek, Joan H. Schiller

**Affiliations:** 1 Psychology Department, University of Virginia, Charlottesville, Virginia, United States of America; 2 Genentech, Inc., South San Francisco, California, United States of America; 3 Division of Hematology and Oncology Division, University of Texas Southwestern, Dallas, Texas, United States of America; 4 Department of Psychology University of Arizona, Tucson, Arizona, United States of America; Geisel School of Medicine at Dartmouth College, UNITED STATES

## Abstract

Societal perceptions may factor into the high rates of nontreatment in patients with lung cancer. To determine whether bias exists toward lung cancer, a study using the Implicit Association Test method of inferring subconscious attitudes and stereotypes from participant reaction times to visual cues was initiated. Participants were primarily recruited from an online survey panel based on US census data. Explicit attitudes regarding lung and breast cancer were derived from participants’ ratings (*n* = 1778) regarding what they thought patients experienced in terms of guilt, shame, and hope (descriptive statements) and from participants’ opinions regarding whether patients ought to experience such feelings (normative statements). Participants’ responses to descriptive and normative statements about lung cancer were compared with responses to statements about breast cancer. Analyses of responses revealed that the participants were more likely to agree with negative descriptive and normative statements about lung cancer than breast cancer (*P*<0.001). Furthermore, participants had significantly stronger implicit negative associations with lung cancer compared with breast cancer; mean response times in the lung cancer/negative conditions were significantly shorter than in the lung cancer/positive conditions (*P*<0.001). Patients, caregivers, healthcare providers, and members of the general public had comparable levels of negative implicit attitudes toward lung cancer. These results show that lung cancer was stigmatized by patients, caregivers, healthcare professionals, and the general public. Further research is needed to investigate whether implicit and explicit attitudes and stereotypes affect patient care.

## Introduction

Lung cancer is the second-most-commonly diagnosed cancer and the leading cause of cancer deaths in the United States. More than 80% of lung cancer deaths are believed to result from smoking; the life-long risk of lung cancer ranges from 10–20% for smokers [1].

An increasing number of studies have shown that more people with advanced lung cancer never receive curative cancer care compared with any other type of cancer patient. In a study of over 700,000 patients with stage IV solid tumors, 24.7% of patients with stage IV non—small-cell lung cancer did not receive anticancer therapy (ie, radiotherapy or systemic therapy) compared with 11.1% of patients with stage IV prostate cancer and 12.8% of patients with stage IV breast cancer. In fact, non—small-cell lung cancer was second only to stage IV kidney cancer (25.2%) for the proportion of patients not receiving anticancer therapy [2]. It is unclear what accounts for the high rate of nontreatment observed in patients with lung cancer. However, emerging research suggests that negative perceptions of lung cancer, such as blame and hopelessness, may play a role [3]. These negative perceptions may be caused by its association with smoking, the thought that the disease is self-inflicted, and its high mortality rate [4]. Consequently, lung cancer carries the cumulative burdens of social stigma and being a leading cause of death [5], and these burdens extend to never-smoking lung cancer patients. Studies have also shown that lung cancer stigma is associated with greater patient-reported symptom severity [6].

Existing studies on the stigma of lung cancer have been limited because they assessed only conscious attitudes, which are partially shaped by social norms. Therefore, to broaden our understanding of lung cancer stigma, we used tests of explicit measures and the Implicit Association Test (IAT), which measures implicit attitudes and beliefs that may exist outside of conscious awareness or conscious control [7].

Project Implicit services have administered more than 16 million sessions measuring implicit associations since its launch in 1998. The robust infrastructure has provided the basis for many dozens of empirical research articles using behavioral measures. The IAT is established as a flexible methodology for measuring a wide variety of associations. The IAT is a well-established tool in social psychological research that measures conceptual associations using reaction time. A substantial literature provides evidence for the adequate reliability, psychometric qualities, and predictive validity of the measure [8–10].

## Materials and Methods

### Study Participants

Participants were recruited from a commercial online survey panel based on US Census data (yougov.com) and by cancer advocacy groups (via newsletters, websites, social media channels, etc.), and included cancer patients, caregivers, healthcare professionals, and members of the general public. As the study was conducted via a website, the only requirement for participation was having access to a computer with Internet access. The study was approved by an institutional review board at the University of Virginia.

### Study Procedure

The study was hosted at www.TheLungCancerProject.org, using software services provided by Project Implicit [11]. Upon visiting the website, participants were first asked a series of questions about their demographic information. Next, participants responded to questions that measured their explicit cancer knowledge, explicit descriptive attitudes, and explicit normative attitudes, in addition to IATs that measured implicit attitudes and stereotypes. The IATs were administered via a Flash application that used the participant’s computer for managing timing of administration and assessment in order to assure accuracy. The entire process took approximately 15 minutes to complete. Upon completion, participants were given feedback about their implicit measures and were provided additional information about the research.

### Explicit Measures

Participants judged themselves as either being very knowledgeable, somewhat knowledgeable, or not at all knowledgeable about cancer. Participants also rated the degree of their agreement with statements such as: “cancer is always fatal,” “cancer is contagious,” “lung cancer is always caused by smoking,” “cancer medicines can help people live longer,” and “early diagnosis can help people live longer” on a six-point scale representing strong, moderate, and slight levels of agreement/disagreement.

Explicit attitudes were measured by recording agreement with descriptive and normative statements. Descriptive statements referenced actual feelings experienced by lung cancer patients and began with: “People with lung cancer are…” The five descriptive statements concluded, respectively, with the phrases: “ashamed about their disease,” “embarrassed to tell others about their disease,” “feel that their own behavior contributed to their disease,” “are likely to die from their disease in a few years,” and “are hopeful about their future” (S1 Table). Normative or prescriptive statements began with: “In my opinion, people with lung cancer ought to be/feel…” The five normative statements used concluding phrases that were identical to the corresponding descriptive items (S2 Table). Despite sharing much of the phrasing, the descriptive “is ashamed” does not imply the normative “ought to be ashamed,” a distinction addressed by the “is—ought” problem in meta-ethics [12]. An identical set of five descriptive and five normative statements were used in the context of breast cancer.

### Implicit Measures

**Fig 1:**
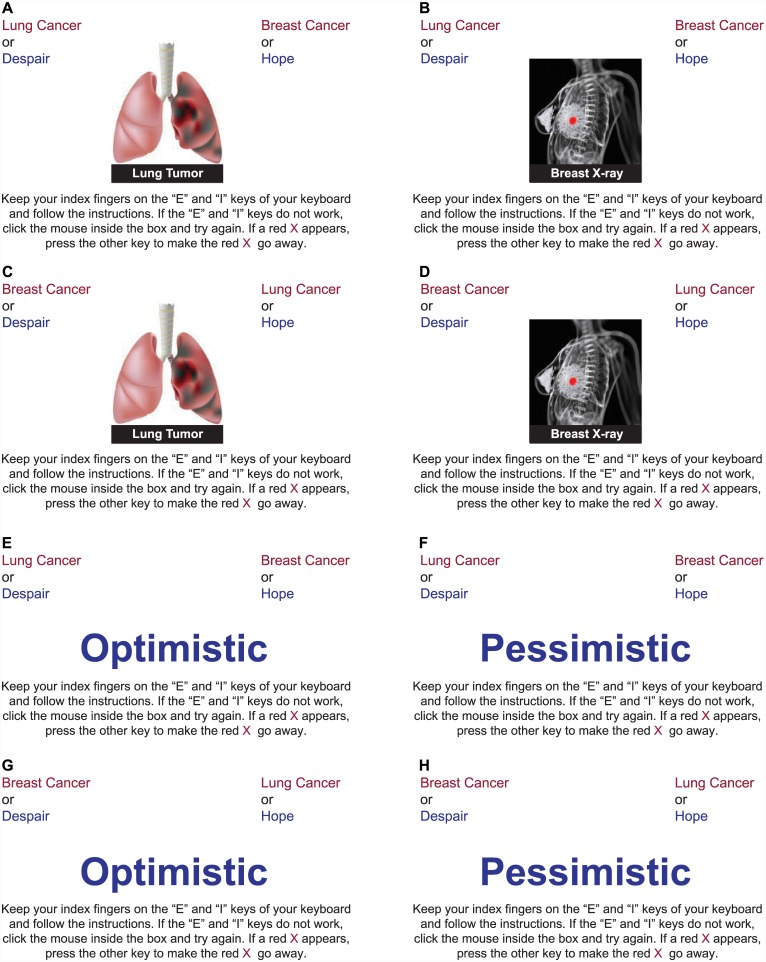
Example of IAT Images (A–D) and Words (E–H) Used in Attitude IATs. IAT, Implicit Association Test.

The premise of the IAT is that concepts associated in memory are categorized rapidly and accurately when they share a common response. If lung cancer is perceived more negatively than breast cancer, participants will categorize items representing lung cancer with items representing negative evaluation (and breast cancer items with positive evaluation) faster than when they categorize lung cancer items with positive evaluation (and breast cancer items with negative evaluation). The strength of the IAT measurement is inferred from the distribution of response times in these two contrasting conditions. In this study, participants were shown a series of images representing either lung cancer or breast cancer (Fig 1), along with words that represented the attitudes good versus bad, hope versus despair, and suitable versus shameful (S3 Table). A second IAT measured the associations between lung cancer or breast cancer and the stereotypes of smoking, drinking, or eating (Fig 1). Participants were randomly prompted to complete one of the three attitude IATs and one of the three stereotype IATs.

As each IAT image or word appeared, participants were asked to either press the “I” key or the “E” key on their keyboard as quickly as possible. In the first block of an attitude IAT, participants responded to images representing “Lung Cancer” (I) or “Breast Cancer” (E), presented in random sequence. In the second block, words representing the categories “Good” (I) and “Bad” (E) were classified.

Seven IAT blocks were used. The first two IAT blocks were for practice, and data were not used in the analysis. The third and fourth blocks combined the mapping rules of the first two blocks. Here, words appeared on odd-numbered attempts and images appeared on even-numbered attempts, in random sequence. In these combined blocks, “Lung Cancer” and “Good” shared the same response key (I) as “Breast Cancer” and “Bad” (E). The fifth IAT practice block was similar to the first block, except that the response mapping for the two cancer concepts were reversed.

In the sixth and seventh IAT blocks, “Breast Cancer” and “Good” shared the same response key (I) as “Lung Cancer” and “Bad” (E). According to the fundamental IAT premise, responses would be faster in blocks 6/7 than in blocks 3/4 if lung cancer was perceived more negatively than breast cancer. The IAT measure was obtained by comparing reaction times in blocks 3 and 4 versus those in blocks 6 and 7. The sign and magnitude of the difference indicate, respectively, the direction and strength of the association. The order of the combined blocks was counterbalanced across participants and the above sequence represents one of the two possible orders. In total, participants completed 7 IAT blocks, 3 of which were for practice and 4 of which were used for data collection and analysis.

Unlike the attitude IATs that assessed implicit valence, the stereotype IATs were varied. In the first two stereotype IATs (“Smoking versus Eating” and “Smoking versus Drinking”), lung cancer was expected to be associated with smoking. The third “Drinking versus Eating” stereotype IAT was not expected to show differential association across the two cancer types.

The IAT effect was computed using the *D* scoring algorithm—the established best practice for analyzing IAT data [13]. The association between lung cancer and negative attributes (for the attitude IATs) or smoking (for the stereotype IATs) was scored as positive (i.e. positive scores indicate faster response times when “lung cancer” was paired with “shameful,” “despair,” or “bad” or when “breast cancer” was paired with “suitable,” “hope,” or “good”). Higher *D* score values indicate a stronger negative association with lung cancer. A sample mean *D* score of 0 could imply either that most participants did not possess implicit associations or that subgroups with associations in opposed directions offset each other. If a sample mean *D* score was negative, that would indicate an association between lung cancer and the positive attribute (or between breast cancer and the negative attribute).

All *p* values reported are two-sided values. All authors had full access to the study data and participated in data analyses.

## Results

A total of 1778 participants were recruited between August 2012 and September 2012 to take part in the study (Table 1); 1051 participants completed the entire procedure and provided complete data on all measures and 727 participants provided data on some, but not all, measures. A majority of participants (1030 [57.9%]) were recruited from the online survey panel at yougov.com. Participants included cancer patients (*n* = 243), caregivers (defined as having cared for or lived with a cancer patient) (*n* = 677), healthcare providers (defined as those who identified themselves as being in the healthcare profession) (*n* = 142), and members of the otherwise general public (*n* = 716).

**Table 1:** Participant Demographics.

Characteristic, No. (%)	Participants (*N* = 1778)
**Method of recruitment**	
Online survey panel	1030 (58)
Advocacy outreach	748 (42)
**Sex**	
Female	1079 (61)
Male	632 (36)
Not identified	67 (4)
**Age**	
Median	50 y
< 30 y	169 (10)
30–49 y	332 (19)
40–49 y	317 (18)
50–59 y	393 (22)
60–69 y	305 (17)
≥70 y	169 (10)
Not identified	93 (5)
**Race**	
White/Caucasian	1362 (77)
Black/African	133 (8)
Hispanic/Latino	123 (7)
East Asian	61 (3)
South Asian	26 (1)
Native Hawaiian/Pacific	12 (1)
Islander	32 (2)
American Indian/Alaska native	29 (2)
Not identified	
**Annual household income**	
Less than $10,000	94 (5)
$10,000–$49,999	568 (32)
$50,000–$99,999	537 (30)
$100,000–$149,999	243 (14)
$150,000–$199,999	119 (7)
$200,000 or more	144 (8)
Not identified	73 (4)
**Highest level of education completed**	
High school or less	440 (25)
Some college	429 (24)
College degree	416 (23)
Graduate study	478 (27)
Not identified	15 (1)
**Occupation**	
Healthcare profession	142 (8)
Nonhealthcare profession	1605 (90)
Not identified	31 (2)
**Cancer diagnosis**	
Yes	243 (14)
No	1497 (84)
Not identified	38 (2)
**Caregiver**	
Yes	677 (38)
No	1073 (60)
Not identified	28 (2)

### Explicit Measures

**Fig 2:**
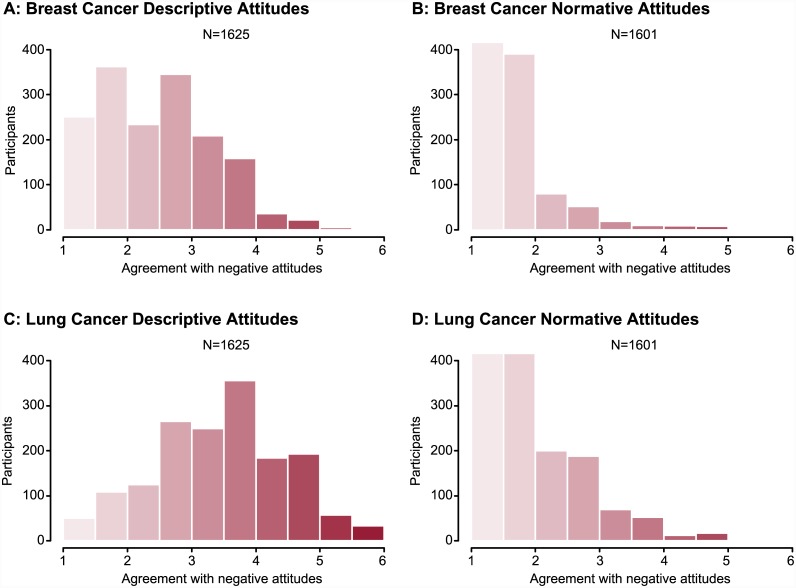
Participant Agreement With Explicit Descriptive Statements (A and C) and Normative Statements (B and D) Toward Breast Cancer (A and B) and Lung Cancer (C and D). Attitudes were scored on a six-point scale, where 1 = strongly disagree, or “negative” and 6 = strongly agree, or “positive”. See S1 and S2 Tables.

Of the 1640 participants who completed the measure of cancer knowledge, 234 participants (14.3%) considered themselves “not at all knowledgeable”, 1104 participants (67.3%) were “somewhat knowledgeable,” and 302 participants (18.4%) noted being “very knowledgeable” about cancer. On the six-point scale (higher scores indicating greater negativity), mean descriptive attitudes toward lung cancer were more negative compared with those toward breast cancer (3.5 versus 2.5; t_1624_ = 37.4, *P*<0.001). The difference in descriptive attitudes correlated positively with self-rated knowledge (r = 0.263, *P*<0.001) (S1 and S2 Tables). Most participants expressed positive normative attitudes toward both cancers by disagreeing strongly with most of the normative negative statements. Despite this, the mean normative attitude was more negative toward lung cancer compared with breast cancer (1.93 versus 1.49; t_1600_ = 28.2, *P*<0.001) (Fig 2). The difference in normative attitudes was uncorrelated with self-rated knowledge (r = 0.022; *P* = 0.39).

### Implicit Measures

**Fig 3:**
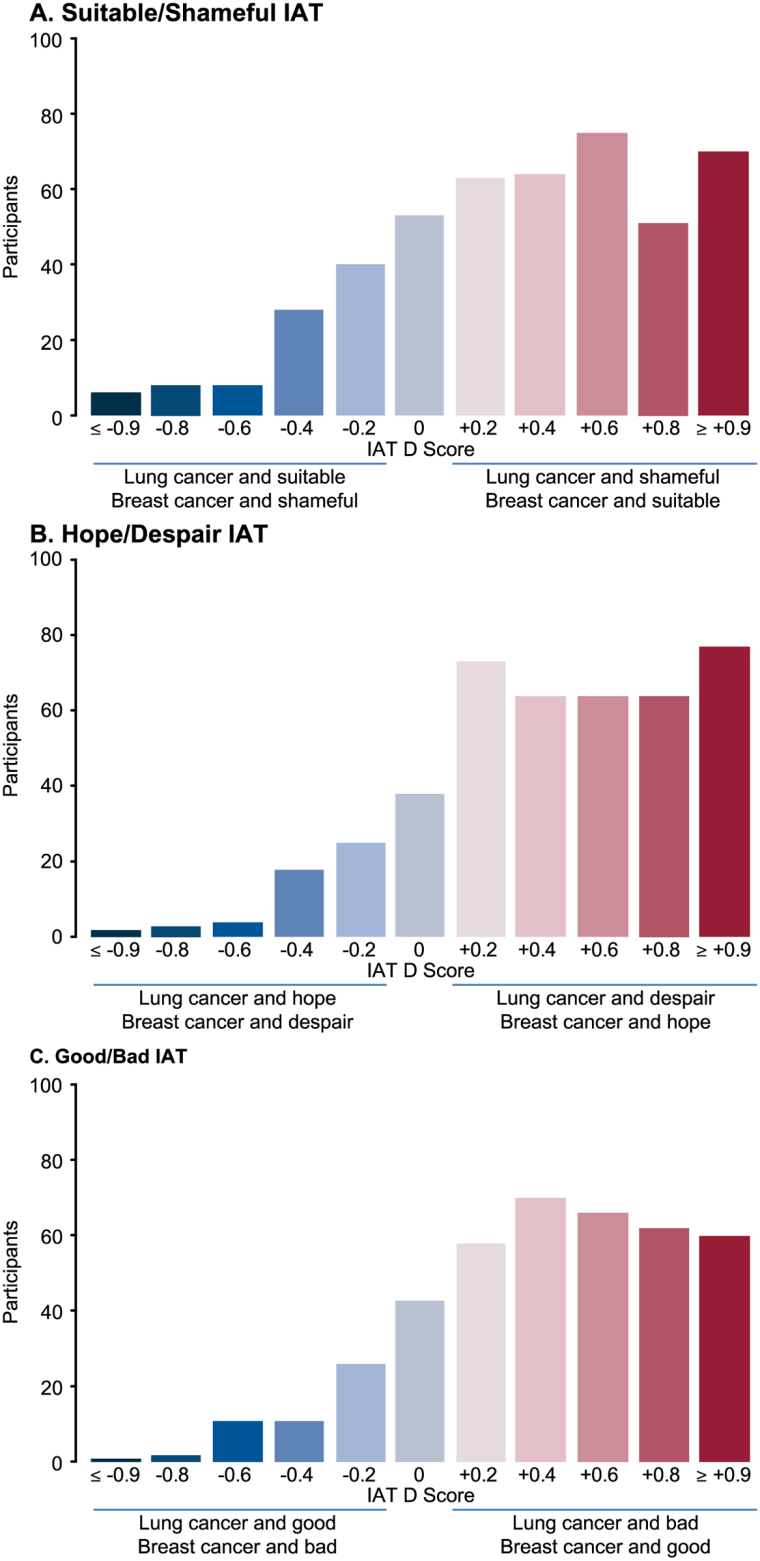
Distribution of IAT D Score Values for Participants Who Completed the (A) Suitable/Shameful IAT, (B) the Hope/Despair IAT, and (C) the Good/Bad IAT. Positive D scores correspond to implicit associations between lung cancer and negative attitudes compared with breast cancer (i.e. faster sorting of “lung cancer” with the negative attribute or of “breast cancer” with the positive attribute). Higher D score values correspond to stronger associations. A D score of 0 indicates that no association exists, and negative D scores correspond with implicit associations of lung cancer with the positive attributes or with breast cancer and the negative attributes. IAT, Implicit Association Test.

In the attitude IATs, participants were faster to respond when a lung cancer image was paired with a negative concept (bad, despair, or shameful) and when a breast cancer image was paired with a positive concept (good, hope, or suitable) than vice versa. The mean response times in the lung cancer/negative conditions were 1011 ms, 1053 ms, and 1107 ms, which were significantly shorter than in the lung cancer/positive conditions at 1195 ms, 1256 ms, and 1266 ms (*P*<0.001 for each). The mean D scores for good/bad (.43), hope/despair (.45), and suitable/shameful (.35) (Table 2) were each considered to represent a strong association between lung cancer and the negative attitude. These associations were also demonstrated by the overall distribution of D score values for participants who completed each IAT (Fig 3). Seventy-four percent of participants (302/410) who completed the good/bad IAT implicitly associated lung cancer with attitude negativity, compared with 10% of patients (41/410) for breast cancer (*P*<0.001); 75% of study participants (325/432) who completed the hope/despair IAT implicitly associated lung cancer with despair, compared with 9% (39/432) for breast cancer (*P*<0.001); and 66% (308/466) of participants who completed the suitable/shameful IAT implicitly associated lung cancer with shame, compared with 17% (81/466) for breast cancer (*P*<0.001) (see Table 2). Cancer patients, caregivers, healthcare providers, and members of the general public all had similar levels of negative implicit bias towards lung cancer (see Table 2).

**Table 2:** Percentage of Participants With Negative Explicit Attitudes, Implicit Attitudes, and Implicit Stereotypes Toward Lung Cancer, Breast Cancer, or Neither.

	All Participants	Caregivers	Patients	Healthcare Providers	General Public
**Negative Explicit Attitudes**					
**Descriptive attitudes** ^a^					
LC bias, %	70	75	81	88	74
BC bias, %	8	7	7	5	8
No bias, %	22	18	12	7	18
**Normative attitudes** ^b^					
LC bias, %	56	59	64	65	56
BC bias, %	3	3	2	3	3
No bias, %	4	38	34	32	41
**Negative Implicit Attitudes**					
**Bad** ^c^					
LC bias, %	74	73	72	63	74
BC bias, %	10	12	13	17	9
No bias, %	16	15	15	20	17
Mean IAT D score	0.43	0.43	0.33	0.33	0.44
**Despair** ^d^					
LC bias, %	75	73	76	77	77
BC bias, %	9	10	5	13	8
No bias, %	16	17	19	10	15
Mean IAT D score	0.46	0.43	0.54	0.44	0.47
**Shameful** ^e^					
LC bias, %	66	65	82	72	66
BC bias, %	17	18	9	11	17
No bias, %	16	17	9	17	17
Mean IAT D score	0.35	0.32	0.52	0.41	0.35
**Implicit Stereotypes**					
**Smoking versus Eating** ^f^					
LC bias, %	86	89	90	94	84
BC bias, %	7	5	5	3	8
No bias, %	7	6	5	3	8
Mean IAT D score	0.62	0.69	0.71	0.79	0.57
**Smoking versus Drinking** ^g^					
LC bias, %	67	65	54	81	69
BC bias, %	18	18	31	5	17
No bias, %	15	17	15	14	14
Mean IAT D score	0.34	0.34	0.17	0.54	0.34
**Drinking versus Eating** ^h^					
LC bias, %	47	40	54	59	50
BC bias, %	29	33	23	29	27
No bias, %	24	27	23	12	23
Mean IAT D score	0.15	0.10	0.22	0.21	0.16

BC, breast cancer; IAT, Implicit Association Test; LC, lung cancer.

All D scores were significantly >0 with *P*<0.001.

^a^ *n* = 1625.

^b^ *n* = 1601.

^c^ *n* = 432.

^d^ *n* = 410.

^e^ *n* = 466.

^f^ *n* = 464.

^g^ *n* = 441.

^h^ *n* = 417.

In the stereotype IATs, participants responded significantly faster when lung cancer and smoking shared a common response, at an average of 968 ms for the smoking/eating IAT and 1057 ms for the smoking/drinking IAT, compared with when breast cancer and smoking were paired together, at 1277 ms and 1222 ms, respectively (*P*<0.001 for each). The mean D scores for the smoking/eating and smoking/drinking IATs were 0.62 and 0.34, respectively, which indicated a strong association between lung cancer and smoking stereotypes. The drinking/eating IAT had a modest mean D score of 0.15, indicating that lung cancer was more strongly associated with drinking than with eating (see Table 2); while this association was statistically significant (*P*<0.001), the strength of the association was weaker than those of the smoking IAT stereotypes.

Implicit attitudes and implicit smoking stereotypes were not correlated with each other, nor were implicit attitudes correlated with the knowledge measures or with the descriptive and normative attitudes. However, the smoking/eating stereotype IAT was significantly correlated with relative descriptive attitudes (r = 0.18, *P*<0.001) and was weakly correlated with relative normative attitudes (r = 0.10, *P* = 0.03). The self-knowledge measure was weakly correlated with the smoking/eating IAT (r = 0.12, *P* = 0.01) and with the smoking/drinking IAT (r = 0.10, *P* = 0.04).

## Discussion

In this study, implicit measures of attitudes and stereotypes associated with lung cancer and breast cancer were measured using the IAT, a well-established tool that has been validated across numerous behavioral, judgment, and psychological contexts [14]. The IAT has been widely used in the field of social psychology to identify implicit social cognition on topics including stigma in mental disease [15], gender stereotypes in science [16], and bias associated with obesity [17]. To our knowledge, this study is the first to use IAT testing to measure societal bias against lung cancer. Now that these biases have been documented, two next obvious steps for research are (1) to examine their implications for judgment and/or behavior (such as treatment decisions, likelihood of joining an advocacy movement, likelihood of seeking treatment), and (2) to investigate methods for changing such biases. Lai and colleagues have identified methods, including evaluative conditioning, for reducing implicit prejudices [18,19]. Whether such methods would have a positive effect on mitigating the implicit biases associated with lung cancer is unknown.

Previous studies of shame and stigma associated with lung cancer have observed that lung cancer stigma can have significant effects on treatment decisions and how patients perceive the severity of their symptoms [3,6]. In this study, both descriptive and implicit measures observed greater levels of shame and stigma associated with lung cancer compared with breast cancer. That these differences were also evident among the healthcare providers and caregivers suggests that patient care could be affected by biases and stigma even when attitudes and/or stereotypes are subconscious [20].

Insights from this study may be important for the development of educational and advocacy efforts to improve perceptions and attitudes toward lung cancer; with medical advances and societal shifts, disease perceptions can change. Breast cancer, which is among the leading causes of cancer deaths among women [21], was once vehemently stigmatized [22]. Breast cancer advocacy has resulted in screening initiatives, educational campaigns, the popularization of the pink ribbon logo as a symbol of hope and solidarity for the afflicted, and has elevated overall awareness of the disease. Perhaps by undertaking a similar partnership-based approach, advocacy groups could begin to reduce the stigma associated with lung cancer. Other potential advocacy-based approaches for reducing lung cancer stigma include education of smoking as an addiction and of lung cancer prevalence in nonsmokers. Increased clinical trial participation among lung cancer patients may show benefit from lung cancer advocacy programs designed in part to reduce shame and stigma.

A limitation of the current study is that the negative bias toward lung cancer may derive primarily from disease features (such as incidence, prevalence, and mortality rates), and the observed difference in bias between the two cancer types may reflect the lower 5-year survival rate of lung cancer compared with breast cancer [23]. Thus, greater knowledge about lung cancer may actually increase negative associations with the disease. Indeed, participants who had more knowledge about lung cancer in this study exhibited more attitude negativity and less hope than those with less knowledge. This dynamic may have important implications for healthcare professionals. Additionally, unintentional selection bias may have influenced the study results, since participants took part voluntarily; while the online survey panel recruited patients based on US Census data, participants were incentivized by rewards to complete online surveys on a number of topics, including the study described here. Lastly, while the IAT has been previously validated by several published studies, the IAT images and word pairs used in the current study have not been used previously and were not independently validated.

The long-term goal of this research is to reduce the stigmatization of lung cancer and to reduce any effect that it may have on whether patients receive anticancer therapy. Future studies are needed to investigate the emergence of attitudes and stereotypes pertaining to lung cancer and their potential effect on care and treatment decisions. Additionally, a reduction in bias may result from increased awareness of lung cancer, such as its incidence in former- and never-smokers, and of newly available treatment options. A combination of these factors has the potential to enhance support for lung cancer patients and ultimately increase the number of patients who receive treatment.

## Supporting Information

S1 Table Descriptive Statements and Results. (DOCX)

S2 Table Normative Statements and Results. (DOCX)

S3 Table Words Used for IAT Attribute Pairs. (DOCX)
