# Supplementary material for: Attitudes and Stereotypes in Lung Cancer versus Breast Cancer
Source: PLoS One. 2015 Dec 23;10(12):e0145715. doi: 10.1371/journal.pone.0145715 (PMC4689531; doi:10.1371/journal.pone.0145715)
Supplement: S1 Table — (DOCX) [file pone.0145715.s001.docx]

**S1 Table. Descriptive Statements and Results.**

|  | **Strongly Disagree** | **Moderately Disagree** | **Slightly Disagree** | **Slightly Agree** | **Moderately Agree** | **Strongly Agree** | **No Answer** |
| --- | --- | --- | --- | --- | --- | --- | --- |
| People with lung cancer are ashamed about their disease. | 371 (20.9%) | 276  (15.5%) | 263 (14.8%) | 421  (23.7%) | 221  (12.4%) | 69  (3.9%) | 157 |
| People with breast cancer are ashamed about their disease. | 581 (32.7%) | 334  (18.8%) | 269 (15.1%) | 258  (14.5%) | 125  (7.0%) | 50  (2.8%) | 161 |
| People with lung cancer are embarrassed to tell others about their disease. | 332 (18.7%) | 296  (16.6%) | 275 (15.5%) | 413  (23.2%) | 224  (12.6%) | 79  (4.4%) | 159 |
| People with breast cancer are embarrassed to tell others about their disease. | 445 (25.0%) | 365  (20.5%) | 264 (14.8%) | 320  (18.0%) | 161  (9.1%) | 65  (3.7%) | 158 |
| People with lung cancer feel that their own behavior contributed to their disease. | 122  (6.9%) | 158  (8.9%) | 212 (11.9%) | 513  (28.9%) | 472  (26.5%) | 139  (7.8%) | 162 |
| People with breast cancer feel that their own behavior contributed to their disease. | 684 (38.5%) | 418  (23.5%) | 272 (15.3%) | 153  (8.6%) | 61  (3.4%) | 23  (1.3%) | 167 |
| People with lung cancer are likely to die from their disease within a few years. | 132  (7.4%) | 209  (11.8%) | 300 (16.9%) | 407  (22.9%) | 374  (21.0%) | 196  (11.0%) | 160 |
| People with breast cancer are likely to die from their disease within a few years. | 378 (21.3%) | 555  (31.2%) | 366 (20.6%) | 185  (10.4%) | 98  (5.5%) | 26  (1.5%) | 170 |
| People with lung cancer are hopeful about their future. | 161  (9.1%) | 340  (19.1%) | 396 (22.3%) | 358  (20.1%) | 233  (13.1%) | 125  (7.0%) | 165 |
| People with breast cancer are hopeful about their future. | 42  (2.4%) | 112  (6.3%) | 206 (11.6%) | 432  (24.3%) | 550  (30.9%) | 274  (15.4%) | 162 |
