# Supplementary material for: Attitudes and Stereotypes in Lung Cancer versus Breast Cancer
Source: PLoS One. 2015 Dec 23;10(12):e0145715. doi: 10.1371/journal.pone.0145715 (PMC4689531; doi:10.1371/journal.pone.0145715)
Supplement: S2 Table — (DOCX) [file pone.0145715.s002.docx]

**S2 Table. Normative Statements and Results.**

|  | **Strongly Disagree** | **Moderately Disagree** | **Slightly  Disagree** | **Slightly Agree** | **Moderately Agree** | **Strongly Agree** | **No Answer** |
| --- | --- | --- | --- | --- | --- | --- | --- |
| In my opinion, people with lung cancer ought to be ashamed about their disease. | 1283  (72.2%) | 161  (9.1%) | 84  (4.7%) | 43  (2.4%) | 11  (0.6%) | 14  (0.8%) | 182 |
| In my opinion, people with breast cancer ought to be ashamed about their disease. | 1461  (82.2%) | 68  (3.8%) | 41  (2.3%) | 10  (0.6%) | 5  (0.3%) | 11  (0.6%) | 182 |
| In my opinion, people with lung cancer ought to be embarrassed to tell others about their disease. | 1342  (75.5%) | 119  (6.7%) | 77  (4.3%) | 33  (1.9%) | 12  (0.7%) | 12  (0.7%) | 183 |
| In my opinion, people with breast cancer ought to be embarrassed to tell others about their disease. | 1420  (79.9%) | 83  (4.7%) | 55  (3.1%) | 10  (0.6%) | 10  (0.6%) | 10  (0.6%) | 190 |
| In my opinion, people with lung cancer ought to feel that their own behavior contributed to their disease. | 701  (39.4%) | 291  (16.4%) | 202  (11.4%) | 260 (14.6%) | 106  (6.0%) | 36  (2.0%) | 182 |
| In my opinion, people with breast cancer ought to feel that their own behavior contributed to their disease. | 1247  (70.1%) | 174  (9.8%) | 109  (6.1%) | 37  (2.1%) | 11  (0.6%) | 10  (0.6%) | 190 |
| In my opinion, people with lung cancer ought to feel likely to die from their disease within a few years. | 824  (46.3%) | 259  (14.6%) | 215  (12.1%) | 162  (9.1%) | 96  (5.4%) | 34  (1.9%) | 188 |
| In my opinion, people with breast cancer ought to feel likely to die from their disease within a few years. | 1009  (56.7%) | 304  (17.1%) | 157  (8.8%) | 54  (3.0%) | 34  (1.9%) | 20  (1.1%) | 200 |
| In my opinion, people with lung cancer ought to be hopeful about their future. | 106  (6.0%) | 110  (6.2%) | 192  (10.8%) | 312 (17.5%) | 378  (21.3%) | 493  (27.7%) | 187 |
| In my opinion, people with breast cancer ought to feel hopeful about their future. | 84  (4.7%) | 44  (2.5%) | 87  (4.9%) | 184 (10.3%) | 488  (27.4%) | 686  (38.6%) | 205 |
