# Supplementary material for: Attitudes and Stereotypes in Lung Cancer versus Breast Cancer
Source: PLoS One. 2015 Dec 23;10(12):e0145715. doi: 10.1371/journal.pone.0145715 (PMC4689531; doi:10.1371/journal.pone.0145715)
Supplement: S3 Table — (DOCX) [file pone.0145715.s003.docx]

**S3 Table. Words Used for IAT Attribute Pairs.**

| **Good Words** | **Bad Words** | **Hope Words** | **Despair Words** | **Suitable Words** | **Shameful Words** |
| --- | --- | --- | --- | --- | --- |
| Wonderful | Dreadful | Confident | Cursed | Appropriate | Disgraceful |
| Good | Bad | Hope | Despair | Suitable | Shameful |
| Excellent | Awful | Cheerful | Dejected | Good | Bad |
| Great | Terrible | Optimistic | Pessimistic | Acceptable | Embarrassing |
